# Lysophosphatidic Acid Enhances Vascular Endothelial Growth Factor-C Expression in Human Prostate Cancer PC-3 Cells

**DOI:** 10.1371/journal.pone.0041096

**Published:** 2012-07-20

**Authors:** Chuan-En Lin, Shee-Uan Chen, Chu-Cheng Lin, Chi-Hao Chang, Yueh-Chien Lin, Yu-Ling Tai, Tang-Long Shen, Hsinyu Lee

**Affiliations:** 1 Institute of Zoology, National Taiwan University, Taipei, Taiwan, Republic of China; 2 Department of Obstetrics and Gynecology, National Taiwan University, Taipei, Taiwan, Republic of China; 3 Department of Life Science, National Taiwan University, Taipei, Taiwan, Republic of China; 4 Department of Plant Pathology and Microbiology, National Taiwan University, Taipei, Taiwan, Republic of China; 5 Center for Biotechnology, National Taiwan University, Taipei, Taiwan, Republic of China; 6 Angiogenesis Research Center, National Taiwan University, Taipei, Taiwan, Republic of China; University of Illinois at Chicago, United States of America

## Abstract

Clinical evidence suggests that lymphangiogenesis and lymphatic metastasis are important processes during the progression of prostate cancer. Vascular endothelial growth factor (VEGF)-C was shown to be a key regulator in these processes. Our previous studies demonstrated that lysophosphatidic acid (LPA), a low-molecular-weight lipid growth factor, enhances VEGF-C expression in human endothelial cells. We previously demonstrated that the LPA receptor plays an important role in lymphatic development in zebrafish embryos. However, the effects of LPA on VEGF-C expression in prostate cancer are not known. Herein, we demonstrate that LPA up-regulated VEGF-C expression in three different human prostate cancer cell lines. In PC-3 human prostate cancer cells, the enhancing effects of LPA were mediated through both LPA1 and LPA3. In addition, reactive oxygen species (ROS) production and lens epithelium-derived growth factor (LEDGF) expression were involved in LPA_1/3_-dependent VEGF-C expression. Furthermore, autotaxin (ATX), an enzyme responsible for LPA synthesis, also participates in regulating VEGF-C expression. By interrupting LPA_1/3_ of PC-3, conditioned medium (CM) -induced human umbilical vein endothelial cell (HUVEC) lymphatic markers expression was also blocked. In summary, we found that LPA enhances VEGF-C expression through activating LPA_1/3_-, ROS-, and LEDGF-dependent pathways. These novel findings could potentially shed light on developing new strategies for preventing lymphatic metastasis of prostate cancer.

## Introduction

Prostate cancer is one of the most frequently occurring cancers in males. The progression of highly metastatic prostate cancer involves processes such as loss of cell adhesion, enhanced local invasion, angiogenesis, and lymphangiogenesis [1]. Lymphangiogenesis was recently found to play an important role in prostate cancer metastasis, and vascular endothelial growth factor (VEGF)-C is a major lymphangiogenic regulator. VEGF-C binds to VEGF receptor (VEGFR)-3 and activates lymphangiogenesis-associated signal pathways [2]. Much clinical evidence revealed a correlation between VEGF-C expression and regional lymph node metastasis in prostate cancer [3], [4]. Over-expressing VEGF-C in LAPC-9 prostate cancer cells enhanced tumor lymphatic metastasis [5]. In the CWR22Rv-1 prostate cancer cell line, cells over-expressing VEGF-C more frequently metastasized to the lymph nodes and lungs. However, the rate of tumor growth and angiogenic behavior were not affected by the over-expression of VEGF-C [6]. Wu et al. in 2008 also showed that a VEGF-C ligand trap and VEGFR-3 antibody significantly reduced prostate cancer lymphangiogenesis and metastasis to lymph nodes and distal organs. All those results suggest that lymphangiogenesis mediates prostate cancer metastasis.

Lysophosphatidic acid (LPA) is a low-molecular-weight lipid growth factor that binds to Edg family G-protein-coupled receptors (GPCRs) and regulates multiple cellular functions [7], [8]. LPA is synthesized by enzymatic cleavage of membrane phosphatidic acid. Once an inflammatory response is triggered, LPA is released from platelet and induces multiple cellular responses such as cell migration, proliferation, and wound healing [9]. In addition, cancer cells over-expressing LPA receptors also exhibited increased tumor invasion and metastasis [10]. The switching expression of LPA_1_ and LPA_3_ receptors is found to be associated with prostate cancer development [11]. In prostate cancer cell line, LPA stimulates PC-3 cells motility through LPA_1_ [12]. In addition, LPA protects PC-3 from starvation-derived apoptosis through a nuclear factor (NF)-κB-dependent pathway [13]. All these results suggest that LPA plays important roles in the development and progression of prostate cancer.

Autotaxin (ATX) is a 125-kDa glycoprotein that belongs to the ectonucleotide pyrophosphatase/phosphodiesterase (ENPP) family which has the ability to hydrolyze phosphodiester bonds *in vitro*. In addition, ATX was also reported to possess lysophospholipase D (LysoPLD) enzymatic function, which hydrolyzes lysophosphatidylcholine (LPC) to generate LPA [14], [15]. In ATX-over-expressing transgenic mice, mammary epithelium is sufficient to initiate tumorigenesis and generate highly metastatic cancer [16]. In addition, in clinical prostate cancer studies, high expression levels of ATX were associated with both malignant potentials and poor outcomes [17].

It was reported that after serum deprivation, VEGF-C messenger (m)RNA expression of cancer cells was enhanced by treatment with 10% serum. Those results suggest that there is a serum factor regulating the expression of VEGF-C [18]. In addition, after knocking down zLPA_1_ in zebrafish, embryonic thoracic duct development was defective [19]. In human umbilical vein endothelial cells (HUVECs), LPA can induce tube formation and lymphatic marker expression through LPA_1/3_ [20], [21]_._ Those results suggest that LPA-derived signals are necessary for lymphatic vessel development. However, the roles of LPA in lymphangiogenesis induced by cancer cells remain uncertain.

In our current results, we show that LPA up-regulated VEGF-C mRNA in different human prostate cancer cell lines. By using an LPA_1/3_ antagonist, we demonstrated that these enhancing effects in PC-3 cells were LPA_1_ and LPA_3_ dependent. Moreover, ROS generation and the transcription factor, lens epithelium-derived growth factor (LEDGF), were involved in LPA’s regulation of VEGF-C expression. ATX, an enzyme responsible for LPA generation, also regulated VEGF-C expression and secretion. Using HUVECs, we demonstrate that conditioned media (CM) from PC-3 cells enhanced the expression of lymphatic markers such as Prox-1 and LYVE-1. In addition, pretreatment with Ki16425, LPA_1/3_ antagonist blocked the enhancing effects of these CM. Our results suggest that LPA and ATX regulate VEGF-C expression in prostate cancer cells and it might lead to lymphatic metastasis. Therefore, the blockade of LPA and VEGF-C signaling might be an effective strategy for prostate cancer treatment.

## Results

### LPA Stimulates VEGF-C Expression in Different Prostate Cancer Cell Lines

**Figure 1:**
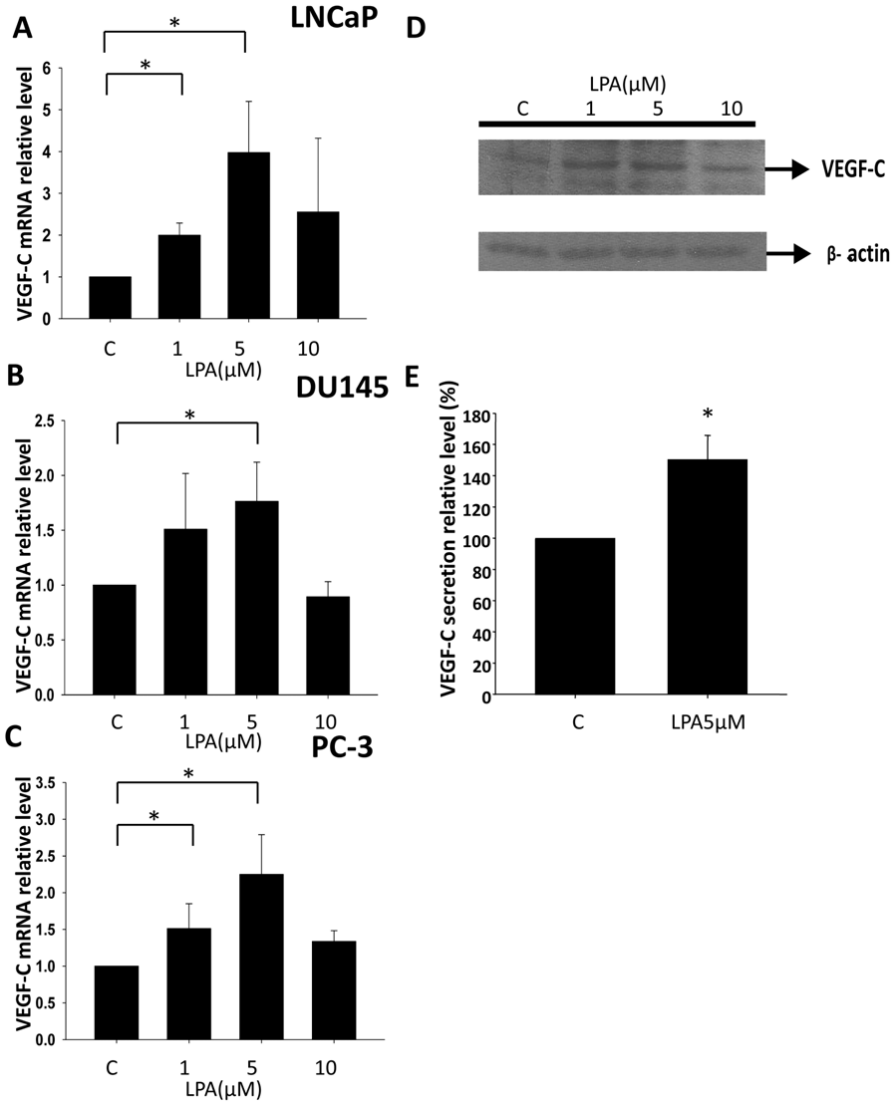
The effect of LPA on prostate cancer cell lines in VEGF-C expression. (A, B and C) The LNCaP, DU145, and PC-3 human prostate cancer cell lines were treated with different LPA dosages for 2 h. mRNA was reverse-transcribed and analyzed by a real-time PCR. The relative level of VEGF-C normalized to GAPDH is shown. (D) PC-3 cells were treated with different concentrations of LPA for 4 h. Cell lysates were resolved by 12% SDS-PAGE and analyzed with Western blotting using an anti-VEGF-C antibody. GAPDH served as the loading control. (E) PC-3 cells were treated with 5 µM LPA for 12 h. The conditioned medium was collected and measured with a VEGF-C ELISA kit. * Statistically different compared to the vehicle-incubated sample treated with LPA (*p<0.05).

To investigate whether LPA is a stimulator for VEGF-C expression in prostate cancer, we selected the LNCaP, DU145, and PC-3 human prostate cancer cell lines to test the possibility. LNCaP is an androgen-dependent and low-metastatic prostate cancer cell line. In contrast, DU145 and PC-3 are both androgen-independent and highly metastatic. All three cell lines were treated with different concentrations of LPA, and RNA samples were collected. From the real-time PCR analysis, we found that LPA stimulated VEGF-C transcription in a dose-dependent manner in all three prostate cancer cell lines (Fig. 1A–C). We used PC-3 cells to further analyze whether LPA enhances VEGF-C protein expression and subsequent secretion. To determine the translational level of VEGF-C, PC-3 cells were treated with LPA, and cell lysates were collected using RIPA buffer. Cell lysates were resolved with SDS-PAGE and detected with a VEGF-C antibody. Intracellular VEGF-C protein levels were enhanced by 1 and 5 µM LPA treatments (Fig. 1D). To determine VEGF-C secretion, PC-3 cells were treated with 5 µM LPA for 12 h, and conditioned media were collected. The conditioned medium was analyzed by a VEGF-C ELISA kit. We found that LPA also stimulates VEGF-C secretion (Fig. 1E).

### LPA-enhanced VEGF-C Expression is Mediated through LPA_1_ and LPA_3_ in PC-3 Cells

**Figure 2:**
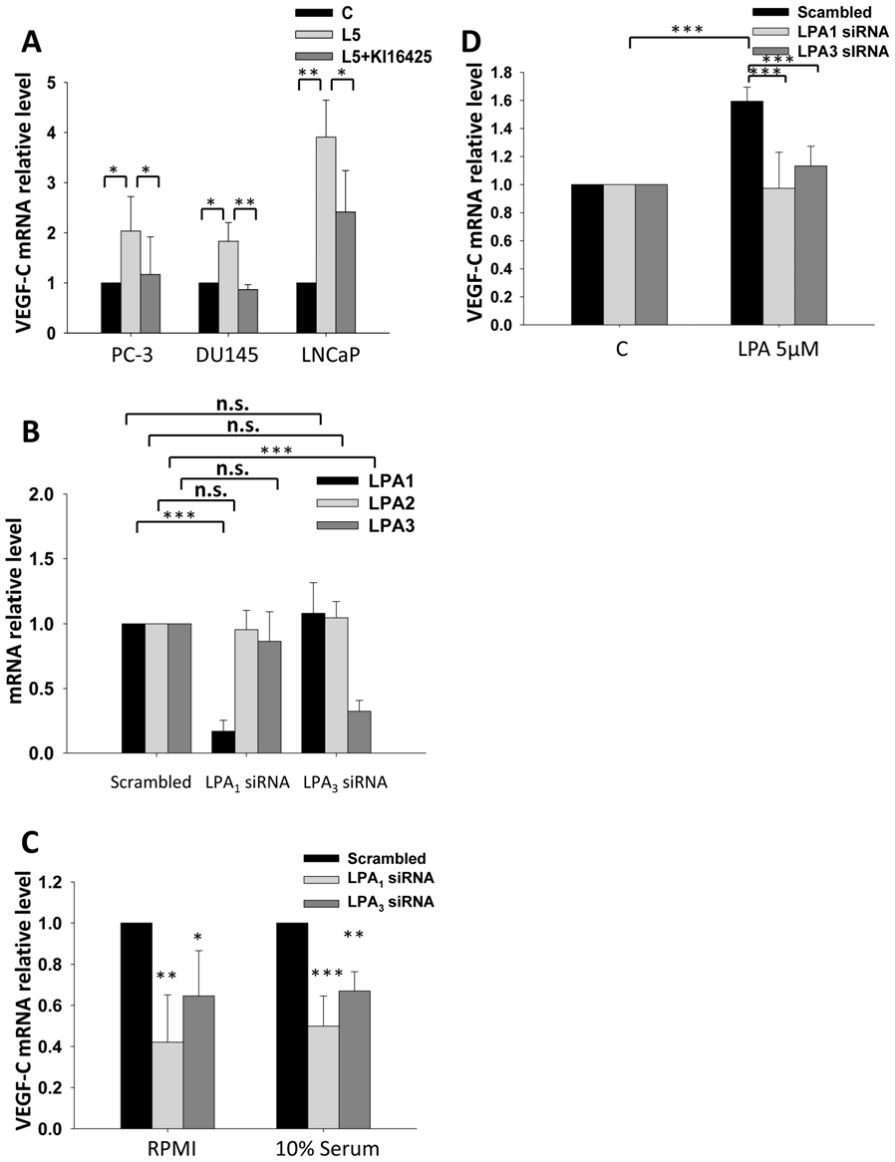
The role of LPA_1_ and LPA_3_ in LPA-enhanced vascular endothelial growth factor (VEGF)-C expression. (A) LNCaP, DU-145 and PC-3 cells were treated with 10 µM Ki16425, an antagonist of LPA_1_ and LPA_3_, followed by 5 µM LPA for 2 h (L5: 5 µM LPA). Relative VEGF-C mRNA levels were measured. (B) PC-3 cells were transiently transfected with LPA_1_ and LPA_3_ siRNA. (C) The basal VEGF-C mRNA expression of PC-3 cells transfected with LPA_1_ or LPA_3_ siRNA was measured in a 10% FBS and no serum conditioned cell culture. (D) PC-3 cells transfected with LPA_1_ and LPA_3_ siRNA were treated with LPA, and VEGF-C mRNA was measured. mRNA was reverse-transcribed and analyzed by a real-time PCR. The relative levels of VEGF-C normalized to GAPDH and β-actin are shown. * Statistically different compared to the vehicle-incubated sample treated with LPA (*p<0.05; **p<0.01; ***p<0.001).

In prostate cancer cells, LPA receptors 1–3 expression profiles are well studied and are thought to associate with prostate cancer development and progression. However, in different prostate cancer cell lines, profiles of different LPA receptors are different. PC-3 cells express the highest LPA_1_ expression level and the lowest LPA_3_ expression level while compared to LNCaP and DU145. In contrast, LNCaP cells express the highest level of LPA_3_ and the lowest level of LPA_1_ among the three prostate cancer cell lines [11]. In our previous study, LPA_1/3_ is responsible for LPA enhanced VEGF-C expression in HUVEC cells [20]. Thus, we first used Ki16425, an antagonist for LPA_1_ and LPA_3_, to determine if these two lysophosphatidic receptors are responsible for LPA effect on VEGF-C expression in prostate cancer cells. In previous study, Ki16425 has already been proved to be able to block ATX induced motility in PC-3 cells [12]. After Ki16425 treatment, the enhancing effect of LPA on VEGF-C expression was decreased significantly in LNCaP, DU145 and PC-3 cells (Fig. 2A). To further confirm the observation, PC-3 cells were transiently transfected with either LPA_1_ or LPA_3_ siRNA. LPA1–3 expression profile was tested in LPA1 and LPA3 transiently knockdown cells. Our results suggested the siRNA sequence we selected could specific target LPA_1_ and LPA_3_ receptor without interrupting the other two receptors (Fig. 2B). Under 10% FBS in RPMI culture, basal mRNA levels of VEGF-C in the LPA_1_ or LPA_3_ knockdown cells were both reduced by 51% and 37% separately. In RPMI only culture, basal mRNA levels of VEGF-C in the LPA_1_ or LPA_3_ knockdown cells were both reduced by 58% and 36% respectively compared to scrambled siRNA transfection control cells (Fig. 2C). This implies that LPA is a major inducer of VEGF-C in serum. Moreover, cell derived LPA might also be a regulator for VEGF-C in PC-3 cells. By direct treatment with LPA, we also observed that LPA-enhanced VEGF-C expression was diminished in PC-3 cells transiently transfected with either LPA_1_ or LPA_3_ siRNA (Fig. 2D). The results suggested that both LPA_1_ and LPA_3_ are involved and critical in controlling VEGF-C expression_._ Besides, there are significant differences among the growth rate of LPA_1_, LPA_3_ knockdown cells and control cells under LPA and serum treatment (Fig. S1).

### LPA_1/3_-enhanced VEGF-C Expression is ROS Dependent

**Figure 3:**
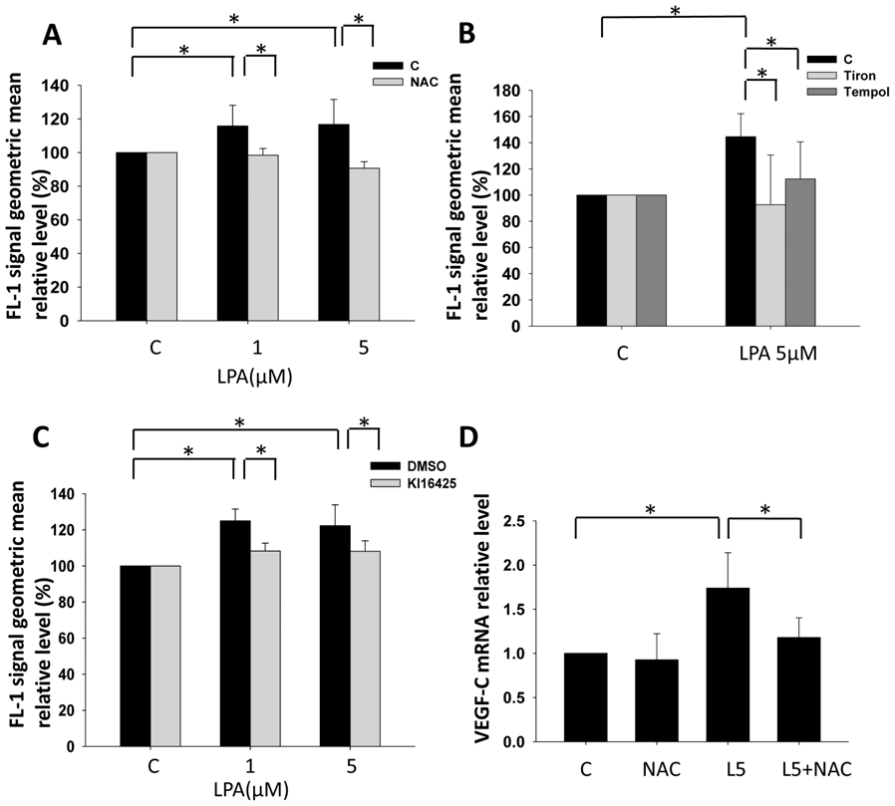
The role of reactive oxygen species (ROS) in LPA_1/3_-enhanced vascular endothelial growth factor (VEGF)-C expression. PC-3 cells were pretreated with 2.5 µM DCFDA for 10 min. Followed by 10 min of LPA stimulation, cells were trypsinized and analyzed by flow cytometry. (A) 10 mM NAC, (B) 1 mM Tiron and 1 mM Tempol were used to treat cell before LPA stimulation. (C) Ki16425 (10 µM) was used to block the LPA_1/3_ signal pathway before LPA stimulation. (D) PC-3 cells were treated with 10 mM NAC, an antioxidant followed by 5 µM LPA for 2 h (L5: 5 µM LPA). The relative VEGF-C mRNA expression was measured. mRNA was reverse-transcribed and analyzed by a real-time PCR. The relative level of VEGF-C normalized to GAPDH is shown. * Statistically different compared to the vehicle-incubated sample treated with LPA (*p<0.05).

After identifying that LPA_1_ and LPA_3_ are involved in VEGF-C expression in prostate cancer cells, we further investigated the possible downstream signaling pathways involved. Since ROS are known to be a VEGF-C inducer [22], [23], we hypothesized that LPA-induced ROS production turns on VEGF-C transcription in PC-3 cells. Using DCFDA as an intracellular ROS detector, we observed that LPA enhanced ROS production in PC-3 cells, and the response could be abolished by pretreatment with N-acetylcysteine (NAC), a treatment known to reduce ROS concentrations (Fig. 3A). Besides NAC, two antioxidants, Tempol and Tiron were also used to confirm LPA induced ROS production (Fig. 3B). Furthermore, LPA-induced ROS were blocked by pretreatment with Ki16425 (Fig. 3C), suggesting that LPA_1/3_ is responsible for ROS generation and the ROS production is not derived from intracellular LPA oxidation. We also observed that LPA-induced VEGF-C was abolished by NAC (Fig. 3D). The results suggested that ROS production is involved in LPA_1/3_-induced VEGF-C expression.

### LPA_1/3_ Mediates VEGF-C Expression through Inducing LEDGF

**Figure 4:**
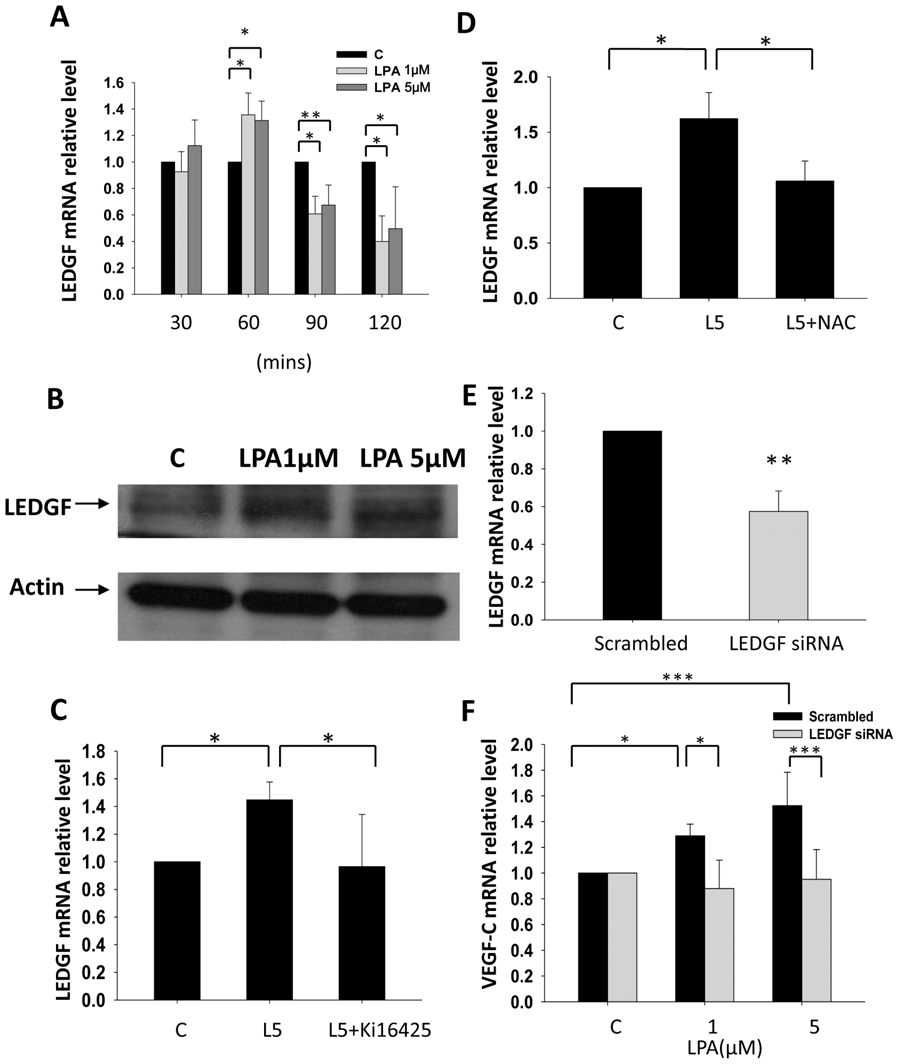
The role of LEDGF in LPA induced VEGF-C expression. PC-3 cells were treated with LPA and mRNA (A) and protein (B) expression levels of LEDGF were determined. LEDGF proteinm was collected after 2 hr LPA treatment. Pretreatment with 10 µM Ki16425 and 10 mM NAC for 1 h suppressed the effects of LPA (L5: 5 µM LPA) on LEDGF expression (C and D). LEDGF siRNA was transfected into PC-3 cells (E) and suppressed LPA’s effects on VEGF-C expression (F). mRNA was reverse-transcribed and analyzed by a real-time PCR. The relative levels of LEDGF and VEGF-C normalized to GAPDH are shown. * Statistically different compared to vehicle-incubated samples treated with LPA (*p<0.05; **p<0.01; ***p<0.001).

Lens epithelium-derived growth factor (LEDGF/p75) was identified as a stress-response protein induced by serum-deprived starvation, thermal stress, and oxidative stress [24], [25]. In prostate cancer, LEDGF was further identified as a drug-resistance gene to attenuate Docetaxel-induced caspase and lysosomal pathways [26]. Recently, oxidative- and thermal stress-induced VEGF-C transcription was found to be mediated by LEDGF in lung carcinoma [22]. Moreover, gonadotropin-regulated lymphangiogenesis is also mediated by LEDGF-induced VEGF-C expression in ovarian cancer [27]. So, we hypothesized that LPA-induced VEGF-C expression is mediated through the LEDGF. Our results demonstrated that LPA induced LEDGF mRNA and protein expression (Fig. 4A, 4B). Moreover, our results demonstrated that LPA induced LEDGF mRNA is transient and quickly declined. The results strongly suggested that a post-transcription mechanism might also be involved in maintaining LPA induced LEDGF protein expression level. Pretreatment with Ki16425 and NAC completely blocked the enhancing effects of LPA on LEDGF expression (Fig. 4C, 4D), suggesting the involvement of LPA_1/3_ receptors and ROS. In addition, LEDGF siRNA was transfected into PC-3 cells to confirm whether LEDGF is involved in LPA-induced VEGF-C expression (Fig. 4E). Results clearly demonstrated that LEDGF is required for the enhancement effect of LPA on VEGF-C expression (Fig. 4F).

### Blockage of LPA_1/3_ Receptor and Knockdown of ATX Expression Reduce VEGF-C Transcription and Secretion by PC-3 Cells

**Figure 5:**
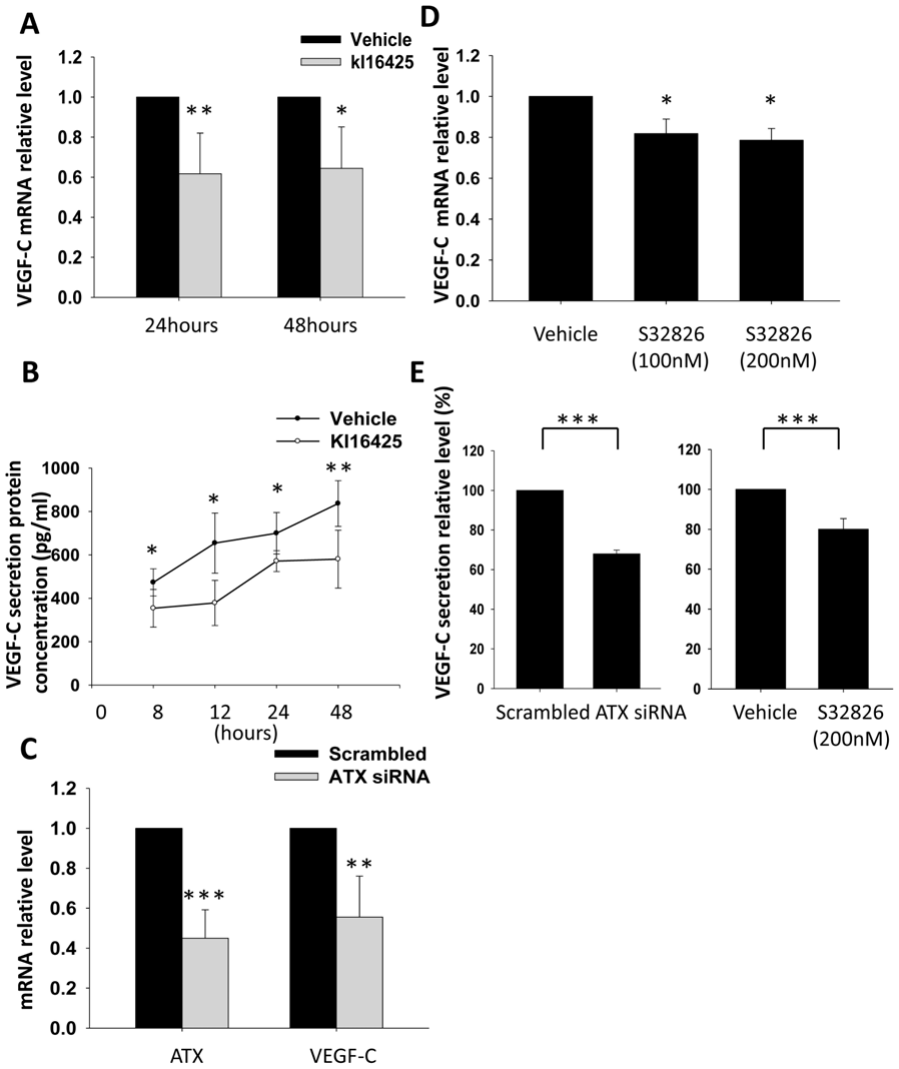
The role of ATX in regulating VEGF-C expression in PC-3 cells. After 16 h of starvation, PC-3 cells were treated with 10 µM Ki16425 in RPMI1640 with 0.005% fatty acid BSA for 8, 12, 24, and 48 h. The relative level (A) and secretion concentration of VEGF-C mRNA (B) in samples were determined. Furthermore, PC-3 cells were transfected with ATX siRNA for 24 h to knock down the ATX gene. PC-3 cells were cultured in RPMI-1640 with 0.005% fatty acid free BSA for another 24 h, and sample RNA was collected. In addition, the inhibitor, S32826, was also used to inactivate ATX activity for 12 h. ATX and VEGF-C mRNA expressions (C and D) were measured. Following the same protocol, PC-3 cells transfected with ATX siRNA and treated with S32826 were starved and cultured in RPMI-1640 with 0.005% fatty acid free BSA for 48 h. VEGF-C secretion concentrations (E) of samples were determined. Sample mRNA was reverse-transcribed and analyzed by a real-time PCR. Relative levels of genes normalized to β-actin are shown. VEGF-C secretion concentrations were measured by an ELISA. * Statistically different compared to the vehicle-incubated sample treated and transfected with Ki16425 and ATX siRNA (*p<0.05; **p<0.01; ***p<0.001).

LPA is highly enriched in serum and platelets; however, many reports demonstrated that breast cancer, glioma, and prostate cancer can self-synthesize LPA and promote tumor progression [28], [29], [30]. ATX is a lysoPLD-hydrolyzing LPC which generates LPA. LPA secreted by ATX increases the invasiveness and progression of breast cancer. In gliomas, lysoPLD activity of ATX also promotes cancer cell adhesion and invasiveness. Based on those previous reports, we were also trying to determine whether prostate cancer cells synthesized ATX could self-regulate VEGF-C expression. Therefore, PC-3 cells were cultured in RPMI-1640-only medium to avoid a serum effect. By blocking LPA_1/3_ with Ki16425, basal VEGF-C mRNA (Fig. 5A) and secretion (Fig. 5B) in PC-3 cells were significantly reduced. These results suggested that LPA secreted by PC-3 cells activated LPA_1/3_ and therefore regulated VEGF-C expression. Furthermore, ATX siRNA and its inhibitor, S32826 [31] (Fig. 5C, 5D), were used to determine whether ATX is involved in regulating VEGF-C. The results demonstrated that down-regulation of ATX gene expression and activities decreased VEGF-C mRNA (Fig. 5C, 5D) and secretion (Fig. 5E). The results suggest that ATX is involved in self-regulating VEGF-C expression in prostate cancer cells.

### By Interrupting LPA_1/3_ of PC-3, Conditioned Medium (CM)-induced HUVEC Lymphatic Marker Expression was Blocked

**Figure 6:**
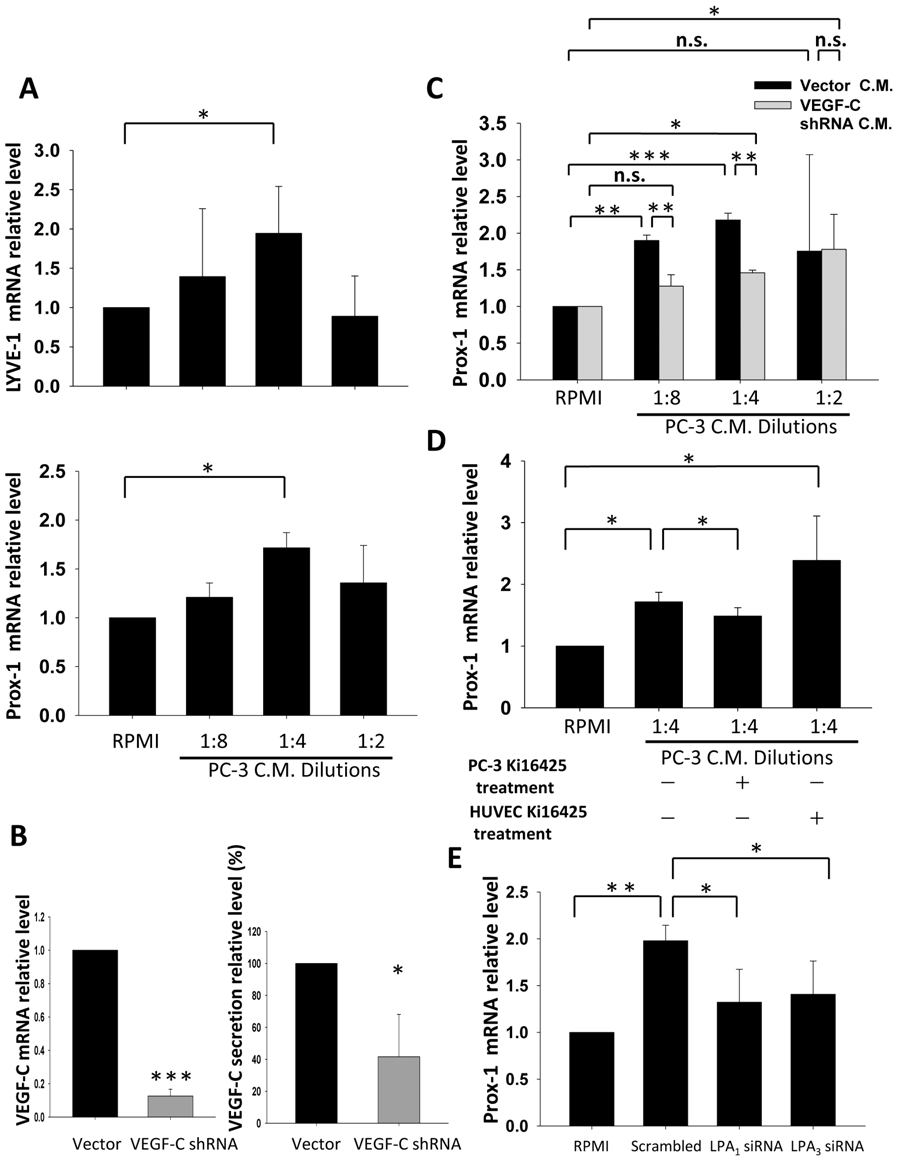
The role of LPA_1_ and LPA_3_ in PC-3 conditioned medium (CM) induced lymphatic marker expression. (A) PC-3 cells were cultured in RPMI-only medium for 24 h after 16 h of starvation. The 24 h CM was collected and diluted to treat HUVECs for 4 h. After treatment, HUVEC lymphatic marker expression was observed. (B) PC-3 cells stably transfected with VEGF-C shRNA were tested for VEGF-C mRNA and secretion expression. (C) VEGF-C knockdown PC-3 CM was used to treat HUVECs following the procedure described above. (D) PC-3 cells were treated with 10 µM Ki16425 while being cultured in RPMI-only medium for 24 h before CM was used to treat HUVECs. Ki16425 was also used to test whether LPA existing in PC-3 CM is crucial for HUVEC lymphatic marker regulation. Ki16425 (10 µM) was pretreated for 1 h before PC-3 CM treatment of HUVECs. (E) CM from PC-3 cells transfected with LPA_1_ or LPA_3_ siRNA was used to treat HUVEC cells as procedures described above. Sample mRNA was reverse-transcribed and analyzed by a real-time PCR. Relative levels of genes normalized to GAPDH and β-actin are shown. * Statistically different compared to vehicle-incubated samples and samples treated with PC-3 CM (*p<0.05; **p<0.01; ***p<0.001).

To mimic the microenvironment between prostate cancer cells and endothelial cells, we used HUVECs as a model to investigate the mechanisms by which prostate cancer cells induce lymphangiogenesis. HUVECs were treated with dilutions of PC-3 CM. The expression levels of lymphatic markers, including Prox-1 and LYVE-1 were up-regulated in HUVECs (Fig. 6A) after CM treatment. To determine whether VEGF-C secreted by prostate cancer cells is required to stimulate lymphatic markers, PC-3 cells stably transfected with VEGF-C shRNA were selected (Fig. 6B). Using CM from VEGF-C knockdown PC-3 cells to treat HUVECs, lymphatic marker expression in HUVECs was significantly reduced compared to vector control group CM (Fig. 6C). This result indicates that VEGF-C is essential for triggering lymphatic marker expression by PC-3 cells. Our results are consistent with previous findings that VEGF-C plays a key role in prostate cancer-induced lymphangiogenesis. Furthermore, we pretreated PC-3 cells with Ki16425 before collecting the CM (Fig. 6D). The results demonstrated that by interrupting the LPA_1/ 3_, the capacity of PC-3 CM to induce lymphatic marker expression was reduced by 32% compared to the control group. However, pretreatment of HUVECs with Ki16425 did not affect the induction of lymphatic marker expression (Fig. 6D), suggesting that the inducing ability in CM is likely due to VEGF-C. For further clarify the roles of LPA_1_ and LPA_3_ in PC-3_,_ we collected conditioned medium from LPA_1_ or LPA_3_ transiently knockdown cell to treat HUVEC cells. In our results, conditioned media from LPA_1_ or LPA_3_ knockdown cells had limited capacity to induce lymphatic marker expression in HUVEC (Fig. 6E).

## Discussion

In this study, we investigated the roles of LPA in regulating VEGF-C expression in prostate cancer. Our results clearly demonstrated that LPA induced VEGF-C expression in different prostate cancer cell lines. LPA_1_ and LPA_3_ were both identified as being involved in the LPA-induced VEGF-C signaling pathway. For downstream signals, LPA-induced ROS production and LEDGF expression were identified as participating in the regulation of VEGF-C. Furthermore, ATX gene knockdown reduced basal VEGF-C expression. This indicates that LPA and ATX are both essential for the regulation of VEGF-C expression in prostate cancer cells.

Lysophospholipids were suggested to promote prostate cancer progression. LPA induces PC3 cell migration through the LPA_1,_ p42, and p38 alpha pathways and also promotes Matrigel invasion [32]. Recently, it also indicate that CD97 heterodimerized and functionally synergized with LPA_1_ implicate in cancer progression [33]. Besides, LPA induced prostate cancer survival and invasion has been shown to associate with calpain-mediated proteolysis of FAK [34]. Moreover, VEGF expression is activated by LPA through activating hypoxia-inducing factor (HIF)-1α expression in PC-3 cells [35]. However, the role of LPA in prostate cancer-induced lymphangiogenesis is still poorly understood.

VEGF-C is considered a key lymphangiogenic factor in initiating lymphangiogenesis and lymphatic metastasis in prostate cancers. In LNCaP cells, VEGF-C was identified to be negatively regulated by androgen through the insulin–like growth factor 1 receptor (IGF-IR) and FOXO pathway [36]. In addition, with androgen ablation, RalA stimulates VEGF-C synthesis by increasing ROS generation [23]. Since RalA activation is triggered by LPA_1_ in HEK293 cells [37], the relationship between LPA receptor activation and androgen’s effects on the regulation of VEGF-C is worthy of further investigation. Our results suggested that both LPA_1_ and LPA_3_ are responsible for LPA-induced VEGF-C expression. Moreover, in LPA_1_ and LPA_3_ stably knocked-down PC-3 cells, basal VEGF-C gene expression was shown to have declined. In our recently published study, LPA-induced VEGF-C and lymphangiogenesis in HUVECs were also LPA_1_ and LPA_3_ dependent [38]. These results suggest that activation of LPA_1_ and LPA_3_ regulates VEGF-C expression in both cancer and endothelial cells.

In prostate cancer, VEGF-C up-regulation was shown to be associated with ROS generation [22], [23]. The results suggested a role of ROS in LPA-induced VEGF-C expression. Our results showed that ROS generation was induced by LPA and participates in LPA-induced VEGF-C transcription. Moreover, LEDGF, a transcription factor activated by ROS, is involved in LPA-induced VEGF-C expression. In clinical research, LEDGF expression was elevated in 93% of clinical prostate tumor samples, and it attenuated docetaxal-induced cell death [39]. Herein, we showed that LEDGF expression was induced by LPA treatment in an LPA_1/3_-dependent manner. These results further support LPA being an important regulator in prostate cancer progression.

Expression levels of ATX, an LPA-producing enzyme, are higher in androgen-independent prostate cancer cells [40]. In addition, clinical data revealed that about 50% of prostate cancer patients showed strong expression of ATX. Moreover, the ATX expression level was significantly correlated with the primary Gleason grade of cancer foci and the presence of prostate capsular invasion [17]. Our results suggest that VEGF-C is regulated by ATX expression in PC-3 cells. Therefore, prostate cancer-synthesizing LPA may form an autocrine loop to stimulate VEGF-C expression and further promote cancer progression. Based on these findings, an ATX inhibitor might be a potential candidate to prevent lymphangiogenesis-induced metastasis of prostate cancer.

Conditioned medium collected from cancer cells is widely used to mimic local microenvironmental interactions between hosts and tumors. Using a HUVEC in vitro model, cancer cells were shown to secret interleukin (IL)-1α to stimulate IL-8 secretion by endothelial cells [41]. Moreover, conditioned medium of the A549 lung cancer cell line regulates endothelial cell platelet derived growth factor (PDGF) expression which is VEGF dependent [42]. All those results suggest that HUVEC cell is a proper *in vitro* model to investigate active components in conditioned medium of cancer cell cultures. PC-3-conditioned medium induced lymphatic endothelial cell (LEC) proliferation, tube formation, and wound healing. Moreover, VEGFR-2 signaling was also identified to play a critical role in LECs’ response to treatment with PC-3-conditioned medium [43]. Herein, we demonstrated that VEGF-C is an important inducer of the expression of HUVEC lymphatic markers in PC-3-conditioned medium. Our results are consistent with previous studies that PC-3 cells stably expressing VEGF-C siRNA reduced intratumoral lymphangiogenesis [44]. Moreover, our research further demonstrated that activation of LPA_1/3_ regulates PC-3 VEGF-C expression, and conditioned medium of PC-3 was capable of inducing the expression of endothelial cell lymphatic markers.

In summary, we show that the enhancing effect of LPA and ATX axis on VEGF-C expression in prostate cancer cells. Therefore, antagonists of LPA_1/3_ and ATX may be feasible therapeutic strategies for inhibiting prostate cancer-induced lymphangiogenesis and therefore prevent metastasis-related cancer deaths.

## Materials and Methods

### Cell Culture

The PC-3, DU145, and LNCaP human prostate cancer cell lines obtained from ATCC were cultured in RPMI (Invitrogen, Carlsbad, CA, USA) supplemented with 10% fetal bovine serum (FBS), penicillin (100 U/ml), and streptomycin (100 U/ml) at 37°C in a humidified atmosphere of 5% CO2. Human umbilical cords were kindly provided by National Taiwan University Hospital (Institutional Review Board approval no. 9561709146). HUVECs were cultured on 1% gelatin-coated (Sigma, St. Louis, MO, USA) 10-cm plates in 60% M199 (Invitrogen, Carlsbad, CA, USA) medium supplemented with 100 U/ml penicillin, 100 mg/ml streptomycin, 20% FBS, and 20% endothelial growth medium (EGM). Cells underwent one passage weekly. Cells were sub-cultured after trypsinization and used in the experiments until passage 4. PC-3 stably transfected with VEGF-C small-hairpin (sh)RNA was maintained and amplified in complete medium supplemented with puromycin (0.25 µg/ml) for 6 weeks before the experiments were performed.

### LPA Stimulation

LPA (Sigma, St. Louis, MO, USA) was prepared in chloroform and methanol (1∶9) and stored at −20°C. One hundred thousand cells were cultured in 3.5-cm-diameter plates with complete medium. After 24 h of implantation, PC-3 cells were starved with serum-free RPMI-1640 for 16 h. After starvation, LPA was added to serum-free RPMI with 0.005% fatty acid-free bovine serum albumin (BSA) as a carrier. The LPA_1/3_ antagonist, Ki16425 (Cayman, Ann Arbor, Michigan, USA) and the ATX inhibitor, S32826 (Echelon, Salt lake city, UT, USA) were both dissolved in DMSO. The working concentration for Ki16425 and S32826 were 10 µM and 200 nM.

### Western Blot Analysis

After treatment, cells were washed with ice-cold phosphate-buffered saline (PBS) and lysed on ice with RIPA buffer (50 mM Tris at pH 8.0, 150 mM NaCl, 1% Triton X-100, 0.5% sodium deoxycholate, and 0.1% sodium dodecylsulfate (SDS)). Lysates were centrifuged at 4°C and 14,000 rpm for 15 min, and clarified supernatants were collected. Equal amounts of samples were separated by 12% SDS-polyacrylamide gel electrophoresis (PAGE) and then transferred to polyvinylidene fluoride membranes (Millipore, Bellerica, MA, USA). The following antibodies were used for blotting: goat monoclonal anti-hVEGF-C antibody (AF752; R&D, Minneapolis, MN, USA), goat polyclonal anti-hLEDGF antibody (sc-33371; Santa Cruz Biotechnology, Santa Cruz, CA, USA), goat polyclonal anti-beta-actin antibody (Santa Cruz), and anti-goat immunoglobulin G (IgG) antibody (HAF109; R&D). Immunoreactive proteins were visualized by enhanced chemiluminescence (Millipore).

### Enzyme-linked Immunosorbent Assay (ELISA)

Starved PC-3 cells were treated with LPA, and conditioned medium was subjected to a VEGF-C ELISA using a kit from R&D Systems (DVEC00). All ELISA procedures followed the manufacturer’s instructions.

### Flow Cytometry

Intracellular ROS were measured by flow cytometry using 2′,7′-Dichlorofluorescin diacetate (DCFDA) (Sigma) as a fluorescence probe. DCFDA is a cell-permeable indictor for reactive oxygen species. Once DCFDA is oxidized, it starts to emit green fluorescent which could be detected in FL-1 channel by flow cytometry. Before PC-3 cells were treated with LPA, cells were washed with PBS and incubated with 2.5 µM DCFDA for 10 min. After DCFDA pretreatment, PC-3 cells were stimulated with LPA for 10 min. Afterwards, cells were detached by trypsinization, centrifuged, and re-suspended in PBS. The fluorescence intensity of DCFDA, as an indicator of the ROS level in different treatments was measured in 10000 cells by Cyflow flow cytometry (Partec, Muester, Germany). All experimental procedures were carried out in dim light.

### RNA Isolation and Reverse-transcription (RT)

Total cellular RNA was extracted from PC-3 cells using the TRIzol reagent (Invitrogen). Complementary DNA was synthesized with 1 µg total RNA using a Toyobo RT-polymerase chain reaction (PCR) kit (Toyobo, Osaka, Japan).

### Quantitative Real-time PCR

A real-time PCR was carried out on an iCycler iQ Realtime detection system (Bio-Rad, Hercules, CA, USA) with SYBR-Green I (Thermo, Rockford, IL, USA) as the fluorescent dye, which enabled real-time detection of PCR products according to the manufacturer’s protocol. Gene-specific primers were used, and the specificity was confirmed by melting-curve detection following the real-time PCR. Cycling conditions were 95°C for 3 min, followed by 40 cycles of 95°C for 30 s, 60°C for 30 s, and 72°C for 30 s. For quantification, the target gene was normalized to the GAPDH internal standard gene. Primers for the real-time PCR were: VEGF-C (F-5′-agtgtcaggcagcgaacaaga-3′ and R-5′-cttcctgagccaggcatctg-3′); LEDGF (F-5′-gggccaaacaaaaagctaga-3′ and R-5′-ttcattgctctccccgttat-3′); LPA_1_ (F-5′-ttcaactctgccatgaacccc-3′ and R-5′-ctaaaccacagagtggtcatt-3′); LPA_2_ (F-5′-gccttcctcatcatggttgtg-3′ and R-5′-agtcatcaccgtcttcattagc-3′); LPA_3_ (F-5′-tcagcaggagtgacacaggcag-3′ and R-5′-ggaagtgcttttattgcagact-3′); ATX (F-5′-acaacgaggagagctgcaat-3′ and R-5′-agaagtccaggctggtgaga-3′); Prox-1 (F-5′-ctcctctgaccagtctgc-3′ and R-5′-ggctctgaaatggataggc-3′); LYVE-1 (F-5′-gcctggtgttgcttctcactt-3′ and R-5′-gtgatccccataattctgcatga-3′) GAPDH (F-5′-aaggtgaaggtcggagtc-3′ and R-5′-tgtagttgaggtcaatgaagg-3′); and β-actin (F-5′-gtaccactggcatcgtgatggact-3′ and R-5′-ccgctcattgccaatggtgat-3′).

### shRNA and Small-interfering (si)RNA Transfection

VEGF-C shRNA (Origene Technology, Rockville, MD, USA ) were transfected into PC-3 cells using lipofectamine 2000 (Invitrogen) for 24 h. After transfection, cells were diluted and selected with puromycin (0.25 µg/ml).

LPA1 (sc-43746), LPA3 (sh37088; Santa Cruz Biotechnology, Santa Cruz, CA, USA), LEDGF (5′-agacagcaugaggaagcgdtdt) and ATX (5′-guggaccaaucuucgacuadtdt) siRNAs (Dharmacon, Lafayette, CO, USA) were also transfected into PC-3 cells for 24 h before conducting the experiment. Gene knockdown efficiency was tested by a real-time PCR and ELISA analysis.

### Statistical Analysis

Data were statistically analyzed using one-way analysis of variance (ANOVA), followed by Fisher’s protected least-significant difference (LSD) test (StatView, Abacus Concept, Berkeley, CA, USA). Each result was obtained from at least three independent experiments, and a value of *p*<0.05 was considered statistically significant.

## Supporting Information

Figure S1 **The** **role** **of LPA_1_ and LPA_3_ in LPA-enhanced cell proliferation.** LPA 5*10^4^ PC-3 cells with LPA_1_ or LPA_3_ knockdown was seeded on the cell culture plate for 24 hr. Before cells were treated with 10% FBS and LPA for cell to proliferate, PC-3 cells were starved in RPMI only medium for 16 hr. After 10% FBS and 5 µM LPA treatment for 24 hr, PC-3 cells number was calculated.
(TIF)
