# Supplementary figures and images for: Lysophosphatidic Acid Enhances Vascular Endothelial Growth Factor-C Expression in Human Prostate Cancer PC-3 Cells
Source: PLoS One. 2012 Jul 20;7(7):e41096. doi: 10.1371/journal.pone.0041096 (PMC3401111; doi:10.1371/journal.pone.0041096)

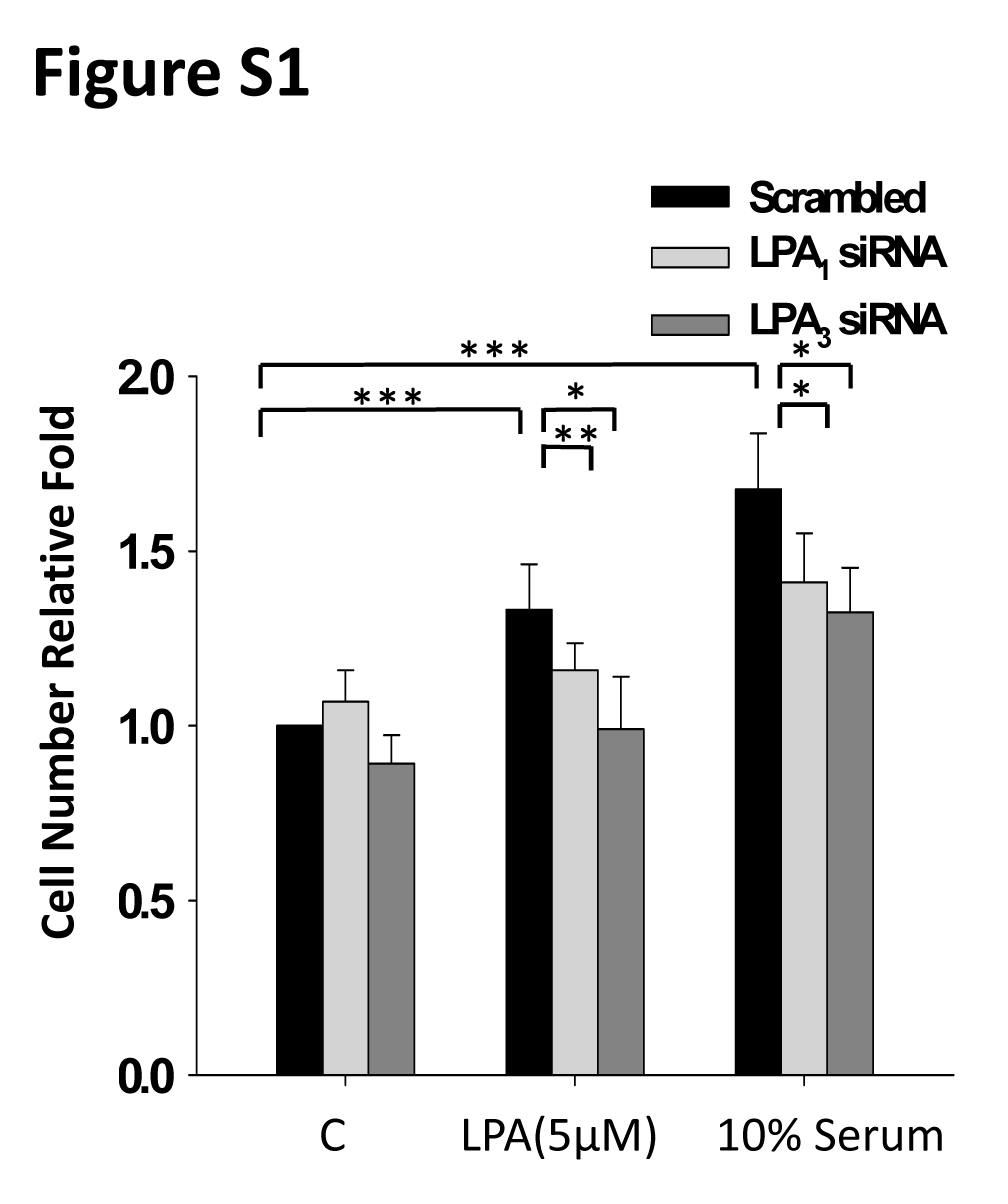

Supplement: Figure S1 — The role of LPA1 and LPA3 in LPA-enhanced cell proliferation. LPA 5*104 PC-3 cells with LPA1 or LPA3 knockdown was seeded on the cell culture plate for 24 hr. Before cells were treated with 10% FBS and LPA for cell to proliferate, PC-3 cells were starved in RPMI only medium for 16 hr. After 10% FBS and 5 µM LPA treatment for 24 hr, PC-3 cells number was calculated. (TIF) [file pone.0041096.s001.tif]
